# Some Causes of Adulterated Food

**Published:** 1912-08

**Authors:** J. S. Abbott

**Affiliations:** Food and Drug Commissioner, Austin, Texas


### Some Causes of Adulterated Food.

BY J. S. ABBOTT, FOOD AND DRUG COMMISSIONER, AUSTIN, TEXAS.

Adulterated food is not always the product of a wicked manufacturer, nor the product of an ignorant manufacturer. However, most food officials and most of our consumers have a contrary opinion. Every food commissioner will, sooner or later, in his career, diligently and studiously seek to discover the real causes of food adulteration. It will not take him long to learn that there are other causes of food adulteration that are as important to be considered as meanness or ignorance on the part of the food manufacturer. The demand of the consumer influences the food manufacturer in the production of his goods in precisely the same way that it does a manufacturer of other articles of commerce. Until he is prohibited by law from doing so, the food manufacturer may be justifiable in yielding to such a demand.

The average housewife selects her foodstuffs almost entirely by the sense of sight rather than with any judgment or knowledge of food values. The sense of taste, even, which plays such an important part in the digestion of food, plays a small part in the selection of foodstuffs.

Nature makes everything beautiful. Dr. Talmage said: "God never made a wave but that He guilded it with a golden sunbeam, nor a star but that He enwreathed it with fern and flower, nor a sky but that He studded it with stars." We admire the beauty of the violet, the blush of the peach, the soft, rich colors of other fruits too numerous to mention. We are not deceived by nature when we regard beauty as a synonym of quality. The deception comes when we extend this faith to what man produces.

The manufacturer is quick to learn what properties control the housewife, in her selection of food, and he is quick and wise to respond. For woe is he who tries to tell a woman she is mistaken about what she wants.

She wants a snowy flour, for some known or unknown reason, and the manufacturer does the most natural thing to be done,—he bleaches it with oxides of nitrogen. She may not be responsible for the origin of the bleaching of flour, but her demand for white flour is the cause of the continuation of the process.

She wants a green English pea, and the Frenchman looks about for a paint of some kind that will give the desired color, or for a chemical that will "fix" the natural green color of the pea, and he finds copper sulphate well adapted for his purpose. She wants her dried fruit to have a sickly, faded, bleached color in place of a rich golden brown, and this is achieved with the aid of the oxides of sulphur. She wants red sausage instead of a natural meat color, and the butcher paints it with Bismarck Brown.

She wants a crisp brittle pickle, and the manufacturer adds alum to it. She knows little and cares less about food values and food substances from which manufactured foodstuffs are prepared for the table. She makes colored, streaked ice cream and cake by using some dye that may injure or impair digestion. Paint hurts the inside as well as the outside of our complexion.

She either wants white rice, or does not know what brown rice is. The natural color of the rice grain is brown. This brown outside covering of the grain, containing much bone and muscle-making material, is scoured off and the grain is coated with a mixture of glucose and talc. This is analogous to the way the wheat grain is treated. Our white flour, as well as our white rice, is composed nearly altogether of starch. Nature fixed the food principles in these grains in the proportion that is most beneficial when used for food. The manufacturer comes along, yielding to the whims of the consumer, and eliminates the very essential elements of diet.

Is it not perfectly reasonable to say that the housewife should spend a little time studying some of our common articles of diet and that she should get some first-hand knowledge of these dietetic value of such foodstuffs and not demand impossible conditions of the product of the food factory?

Another controlling factor in food selection is economy. This is legitimate when used in a legitimate way. It is a factor that we can not squirm around nor dig under. We must, at least most of us must, go straight through it. We buy an article because it is cheap, and never take the trouble to find out what has made it cheap.

A soda water manufacturer sells a case of ginger ale at 40 cents. Another sells it at 50 cents. The one is made of branch water, saccharin, a little ginger and much pepper. The other one uses distilled water, sugar, sure enough ginger, and very little pepper. One baker sells a 14-ounce loaf of bread for 5 cents; another baker sells a 12-ounce loaf of bread for 5 cents. The first loaf may contain 40 per cent moisture and be poorly baked. The other one may contain only 15 per cent moisture and be properly baked. In other words, the high-priced article may have the greatest food value, dollar for dollar. How many housewives have ever seen the dairy that furnishes the supply of milk to her family, or the slaughter house where her meat is butchered?

In other words, do the consumers themselves take any interest in the pure food movement? Do we care whether a baker brings our bread to us in dust-proof paper, or whether he brings it unwrapped in the dirty hands which handle his dirty horses? Does the conscientious manufacturer receive the support of consumers in a way that makes him glad he is living? Does the consumer know, or does he care, whether his favorite soft drink is medicated or not with caffeine, cocaine, formic acid, and the like? Does the housewife care whether or not her groceryman keeps her berries, grapes, and other fruit, out on the sidewalk, exposed, unprotected from flies, dogs, and the manure dust of the streets?

### OTHER CAUSES OF FOOD ADULTERATION.

There are still other causes of food adulteration besides those I have here indicated.

The religious press of our country has been carrying rotten advertising matter of every kind almost. It will not advertise alcoholic beverages, and very properly. But it has advertised quack doctors and quack cure-alls and quack cosmetics, and encouraged the public to diagnose its own ills, and upon that diagnosis to take the secret remedies of some self-designated master-specialist. If you do not believe that this is true, go to your files of our *Baptist Standard,* the *Texas Christian Advocate,* the *Home and State* et al. When I criticised some of the good brethren about this matter, they squirmed and said the present management of this paper was not to blame, that it inherited these advertising contracts, and must carry them out. What would he think if a good brother in his Christian love for your soul were to admonish you to quit your meanness, and you were to tell him that you had to wait a year until the expiration of your contract with the devil?

ANOTHER POTENT CAUSE OF FOOD ADULTERATION.

The weapons, polished and keen, obtainable by the manufacturers, are another potent cause of adulterated foodstuffs. Among these are some of the eminent men of science. Let the Federal government, or a State government, be about to try a big case, and I can name in advance many of these eminent men who will testify for the defendant, no matter what the issue. Especially is this true of some of the educators of our country.

Thank heaven, it is not true of Texas educators, nor of Texas physicians. And may it never be.

A scientific man has a right, and it is perfectly proper for him to do so, to testify for a defendant, provided he sticks to the truth and his testimony is in keeping with the literature he made when writing the facts of his own sake, uninfluenced by court procedure. And a change of mind is legitimate when that change is made by new discoveries of new truths, and not by handsome fees.

In the case of the United States vs. so many barrels of Coca-Cola at Chattanooga, Tennessee, Dr. Vaughan of the University of Michigan said:

"The effect of caffeine in moderate doses is certainly beneficial. Caffeine is no more poisonous than xanthine. Caffeine is xanthine."

In his book on Cellular Toxines he wrote:

"The action of caffeine is directed upon the central nervous system—the muscles and the kidneys. The effects of the former; that is, on the central nervous system, is one of reflex irritability, which, as in the case of strychnine, may lead to complete tetanus and even paralysis."

---

*To My Friends: Will you please send me the name of some nice, bright woman in your town who will make a house-to-house canvass for subscriptions to the "Red Back," on a liberal commission? I want to put it in the homes. If so, please drop me a card at once.* *MRS. F. E. DANIEL.*

THE RECEIPT OF A SAMPLE COPY means: "Your subscription is respectfully solicited."

